# Phenanthroline-Derivative Functionalized Carbon Dots for Highly Selective and Sensitive Detection of Cu^2+^ and S^2−^ and Imaging inside Live Cells

**DOI:** 10.3390/nano8121071

**Published:** 2018-12-19

**Authors:** Lina Zhang, Zhanwei Wang, Jingbo Zhang, Jianbo Jia, Dan Zhao, Yunchang Fan

**Affiliations:** 1 College of Chemistry and Chemical Engineering, Henan Polytechnic University, Jiaozuo 454003, China; 13290700557@163.com (Z.W.); zhangjb5464@163.com (J.Z.); jiajianbo@hpu.edu.cn (J.J.); iamzd@hpu.edu.cn (D.Z.); 2 Henan Key Laboratory of Coal Green Conversion, Jiaozuo 454003, China

**Keywords:** carbon dots, Cu^2+^, S^2−^, (2,3-f)-pyrazino(1,10)phenanthroline-2,3-dicarboxylic acid, fluorescent nanoprobe

## Abstract

Developing effective methods for the instant detection of Cu^2+^ and S^2−^ is highly desired in the biological and environmental fields. Herein, a novel fluorescent nanoprobe was elaborately designed and synthesized by grafting a phenanthroline derivative onto the surface of carbon dots (CDs). The obtained functionalized CDs (FCDs) exhibited blue fluorescence (FL) with excellent photostability and possessed a mean diameter around 4 nm. Cu^2+^ can be selectively captured by the phenanthroline group of FCDs to generate an absorptive complex in situ, leading to obvious quenching of the FCDs’ FL signal through an inner filter effect. Furthermore, the FL of the FCD–Cu^2+^ can be effectively recovered by S^2−^ anions due to the release of FCDs from the FCD–Cu^2+^ complex owing to the formation of stable CuS (*K*_sp_ = 1.27 × 10^−36^) between S^2−^ and Cu^2+^. The detection limits of the FCDs were determined to be 40.1 nM and 88.9 nM for Cu^2+^ and S^2−^, respectively. Moreover, this nanoprobe can also be used for the imaging of intracellular Cu^2+^ and S^2−^, which shows strong application prospects in the field of biology.

## 1. Introduction

Copper ion (Cu^2+^) and sulfide anion (S^2−^) have attracted increasing attention in recent years owing to their vital roles in various pathological/physiological processes and their being highly toxic to the environment and human health [1,2]. As an essential metal in biological systems, copper acts as a cofactor for many enzymes and proteins, and thus abnormal levels of Cu^2+^ may cause neurological problems or damage to livers and kidneys [1]. Meanwhile, Cu^2+^ is also a priority pollutant as listed by the US Environmental Protection Agency (EPA) [3]. Similarly, S^2−^ is a well-known toxic pollutant for the environment [4] and an important anion in biological organisms [5]. Exposure to high concentrations of S^2−^ can induce unconsciousness and suffocation [6], while unbalanced S^2−^ levels in life systems are related to multiple diseases, for instance, Alzheimer’s disease and diabetes [7]. Developing simple and effective methods for the instant detection of Cu^2+^ and S^2−^ is highly desirable in biological and environmental fields.

To date, many methods have been developed for the detection of Cu^2+^ and S^2−^, including electrochemical methods [8,9], inductively coupled plasma atomic emission spectroscopy [10,11], fluorescence (FL) spectrometry [12,13,14], and others. Among those analytical methods, combining FL spectrometry with specific fluorescent sensors has become a promising way to detect Cu^2+^ and S^2−^ because of its non-invasive, economic, real-time, highly selective, and sensitive properties. Recently, various fluorescent sensors have been synthesized for the monitoring of Cu^2+^ or S^2−^ [12,13,14]. However, most of these reported sensors were fabricated on the base of conventional fluorescence materials, such as organic dye molecules or semiconductor quantum dots (QDs) [13,14]. The poor luminescence stability of organic dye molecules [15] and the high toxicity of semiconductor QDs [16] severely restrict their practical applications. Very recently, carbon dots (CDs), as a new family member of carbon nanomaterials, have emerged as promising photoluminescent nanoparticles due to their outstanding properties such as nontoxicity, FL stability, high biocompatibility, water solubility, and facile preparation [17,18]. With these features, CDs exhibit prospective applications in sensing [19,20], FL markers [21], drug delivery [22], and bioimaging [23]. However, it is worth noting that pristine CDs usually show unsatisfactory selectivity or have very limited analytes in sensing applications [7,24,25]. Therefore, it is very important to integrate highly specific recognition molecules into CDs to extend their sensing applications.

**Figure 1:**
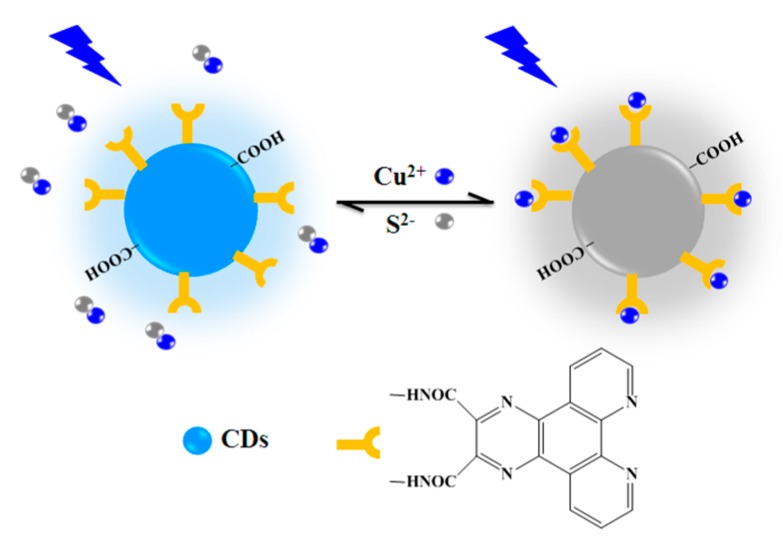
Schematic diagram of functionalized carbon dots (FCDs) for Cu^2+^ and S^2−^ recognition.

We grafted (2,3-f)-Pyrazino(1,10)phenanthroline-2,3-dicarboxylic acid (PPDA), a molecule specific for Cu^2+^ identification [26], onto the surface of carbon dots and provided an effective strategy for the detection and imaging of intracellular Cu^2+^ and S^2−^. The FL interactions between Cu^2+^ and the FCDs were investigated in detail. The phenanthroline groups of the functionalized QDs (FQDs) can selectively capture Cu^2+^ to generate an absorptive complex in situ, which can lead to an intense quenching of the FQDs’ FL through an inner filter effect [24]. Moreover, the FL of the FCD–Cu^2+^ can be effectively recovered by S^2−^ anions due to the release of FCDs from the FCD–Cu^2+^ complex owing to the formation of stable CuS (*K*_sp_ = 1.27 × 10^−36^) (Figure 1) [27]. Furthermore, the FCDs showed remarkable FL stability and can be used for the imaging of intracellular Cu^2+^ and S^2−^. The comparison of several existing CD-based methods for Cu^2+^ or/and S^2−^ detection is listed in Table S1, indicating the good sensitivity and other merits of our sensing system compared with previously reported sensing systems [6,7,27].

## 2. Materials and Methods

### 2.1. Materials

N-Hydroxysuccinimide (NHS, Energy Chemical, Shanghai, China), (2,3-f)-Pyrazino(1,10)phenanthroline-2,3-dicarboxy-lic acid (PPDA, Energy Chemical, Shanghai, China), Ethylenediamine (Energy Chemical), 1-(3-dimethylaminopropyl)-3-ethyl-carbodiimide hydro chloride (EDC, Energy Chemical, Shanghai, China), citric acid (Energy Chemical, Shanghai, China), HEPES (Energy Chemical, Shanghai, China), nitrate salts of metal ion and sodium salts of anion of analytic grade were used as received. HeLa cells (Human cervix carcinoma cell) were purchased from a cell bank in Shanghai (Shanghai, China).

### 2.2. Preparation of CDs

The CDs were prepared according to a modified hydrothermal method [28]. Briefly, 1.0600 g citric acid and 340 μL ethylenediamine were added to 8 mL distilled water under drastically stirring. The generated clear solution was diverted to a Teflon-lined stainless steel vessel (23 mL) and heated at 200 °C for 5 h. Then the product was cooled naturally and subjected to dialysis. Redundant precursors were removed over 24 h via a cellulose membrane (MWCO 500) in pure water. After drying by lyophilization, CD powders were collected (yield: 46%).

### 2.3. Synthesis of PPDA-Functionalized CDs (FCDs)

The ligand PPDA (0.1 mmol) was dispersed in 10 mL of DMSO (Dimethyl sulfoxide), and then 0.2 mmol NHS and 0.2 mmol EDC were added to activate the carboxyl groups of the ligand. The mixtures were stirred for 2 days in the dark. Then, 10 mL aqueous solution of CDs (6 mg/L) was added dropwise to the mixture and the resulting solution was further stirred for 3 days at room temperature. Then, the reaction solution was dialyzed against water for 24 h to obtain the FCDs.

### 2.4. Characterization

The size distribution of the FCDs was measured by a Tecnai G2 F20 TEM characterization (FEI, Hillsboro, OR, USA). Absorption spectra were determined on a Perkin-Elmer-Lambda 900 spectrometer (PerkinElmer, Waltham, MA, USA). FL spectra were measured on a FluoroMax-P spectrophotometer (Horiba Jobin Yvon, Paris, France). Infrared spectra (IR) were measured on a Prestige IR21 FTIR spectrometer (Shimadzu, Kyoto, Japan).

### 2.5. Fluorescence Detection for Cu^2+^ and S^2−^

For the FL detection of Cu^2+^, different amounts of Cu^2+^ (0–50 µM) were added into FCDs dispersed in pH 7.4 HEPES buffered water. Different metal ions (Zn^2+^, Na^+^, Fe^3+^, Mg^2+^, Fe^2+^, Mn^2+^, Ni^2+^, Pb^2+^, Co^2+^, and Cd^2+^) were selected to assess the selectivity of the FCDs towards Cu^2+^.

For the monitoring of S*^2−^*, different amounts of S^2−^ (0–20 µM) were added into a mixture of 20 µM of Cu^2+^ and FCDs dispersed in pH 7.4 HEPES buffered water. Different anions (F*^−^*, Cl*^−^*, HCO_3_^−^, SO_4_^2−^, NO_3_^−^, CO_3_^2−^, and HPO_4_^2−^) were selected to evaluate the selectivity of the FCDs for S^2−^.

The following equation was used to calculate the limit of detection for Cu^2+^ (or S^2−^): the limit of detection = 3*S*_B_/*S*, where *S*_B_ is standard error for the blank test and where *S* is the slope of the calibration curve.

### 2.6. Cell Culture and Fluorescence Imaging

HeLa cells were cultured in RPMI-1640 medium (a class of media developed by Roswell Park Memorial Institut) containing 1% of penicillin-streptomycin and 10% fetal bovine serum (FBS). The cells were grown at 37 °C in an incubator with a 5% CO_2_ atmosphere for 24 h. After removal of the culture medium, cells were incubated with the prepared FCD sensor in 2 mL fresh culture medium at 37 °C for 6 h. Then the medium was removed, and the cells were washed three times with phosphate buffer solution (PBS) to remove the residual nanoparticles. Subsequently, the cells were treated with a certain amount of Cu^2+^ (0, 50, and 100 µM) in 2 mL refresh medium at 37 °C for 1.5 h. After removing the medium and washing it three times with PBS, the cells were suspended in 0.5 mL of PBS and observed under an Olympus FV1000-IX81 laser confocal microscope (Olympus Corporation, Tokyo, Japan).

The S^2−^ imaging experiments were performed using the same procedure as for Cu^2+^, except that cells were first treated using a medium containing the sensor FCD–Cu^2+^ and then by S^2−^ (0, 75, and 150 µM each). Blue emissions from FCDs was excited at 405 nm.

## 3. Results

### 3.1. Synthesis and Characterization of the FCDs

**Figure 2:**
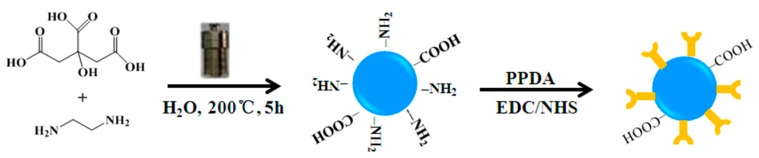
Schematic diagram of the preparation for FCDs.

As portrayed in Figure 2, FCDs were prepared through a modified hydrothermal method and subsequent surface functionalization technique. For this work, PPDA was selected as functional molecules to modify FCDs for two main reasons. Firstly, PPDA can be easily grafted onto the surface of the pristine CDs due to the reaction between the amino group of the CDs and the carboxyl group of PPDA. Secondly, a PPDA with a phenanthroline group has an excellent affinity for Cu^2+^ and has been successfully applied in the selective recognition of Cu^2+^ in a Eu-MOF [26].

**Figure 3:**
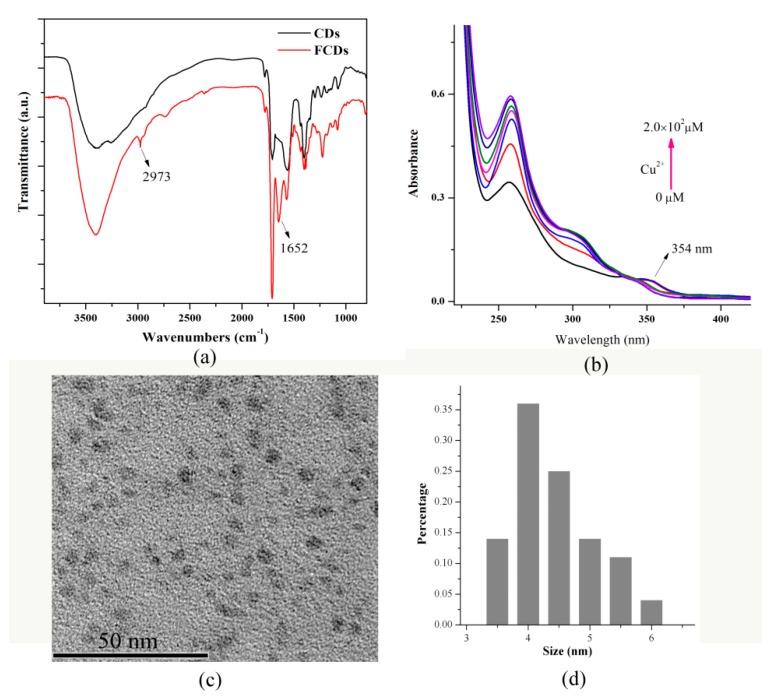
(**a**) IR spectra of carbon dots (CDs) and FCDs; (**b**) UV spectra of FCDs upon addition of Cu^2+^; (**c**) TEM and (**d**) the size distribution histogram of the FCDs.

As demonstrated in Figure S1, the UV-vis absorption spectrum of the pristine CDs exhibited a peak at around 346 nm, while its FL spectra (Figure S2) show a typical excitation-dependent property, demonstrating the synthesis of the desirable CDs [28]. In the IR spectrum (Figure 3a) of the CDs, the peaks observed around 3417, 3248, 1705, 1564, and 1404 cm^−1^ correspond to the O–H stretching, N–H stretching, C=O vibration, N–H bending, and amido C–N stretching vibration, respectively [28]. After grafting PPDA onto the surface of the CDs, new peaks at around 2973 and 1652 cm^−1^ were observed, which correspond to C=C/C=N stretch of polycyclic aromatic hydrocarbons [29]. The appearance of the C=C/C=N stretch in the IR spectrum indicates the formation of PPDA-functionalized CDs.

As shown in Figure 3b, the FCDs exhibit two absorption bands centered at 258 and 354 nm. The band located at 258 nm can be primarily attributed to the π–π* electronic transitions of the ligand PPDA, while the band centered at 354 nm can be ascribed to the absorption of CDs. Upon the addition of Cu^2+^, a new UV absorption band centered at 306 nm appeared and increased gradually, while the absorption intensity of the band centered at 354 nm decreased significantly. These results indicate the interaction between Cu^2+^ and the ligand PPDA and also demonstrate the successful synthesis of the FCDs.

The TEM image (Figure 3c) reveals that the FCDs a1re uniform in morphology with an average diameter of around 4 nm (Figure 3d).

### 3.2. Detection of Cu^2+^ by FCDs

**Figure 4:**
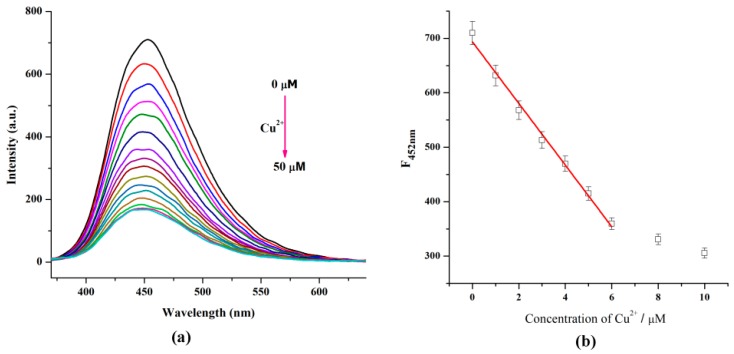
(**a**) Fluorescence (FL) spectra (λ_ex_ = 320 nm) of FCDs upon addition of Cu^2+^; (**b**) Plot of the FL intensity of FCDs against Cu^2+^ concentration, λ_ex_ = 320 nm, *λ*_em_ = 452 nm.

Figure 4a illustrates the FL changes of the FCDs under different concentrations of Cu^2+^. The FL intensity of FCDs clearly decreased with the addition of 5 μM Cu^2+^. For comparison, the FL intensity of the pristine CDs changed a little when 200 μM of Cu^2+^ were added (Figure S3). This clearly shows the interaction between Cu^2+^ and the FCDs, which resulted in the apparent FL decrease of the FCDs. Good linearity between the FL intensity of the FCDs and the Cu^2+^ concentrations was obtained in the range of 0–6.0 µM (Y = 693.4717 − 56.5662X, *R*^2^ = 0.9966, Figure 4b). The detection limit of this sensor was calculated to be 40.1 nM, which is well suited for detecting copper ion in drinking water and blood systems [30].

### 3.3. Mechanism for Cu^2+^ Detection Using FCDs

**Figure 5:**
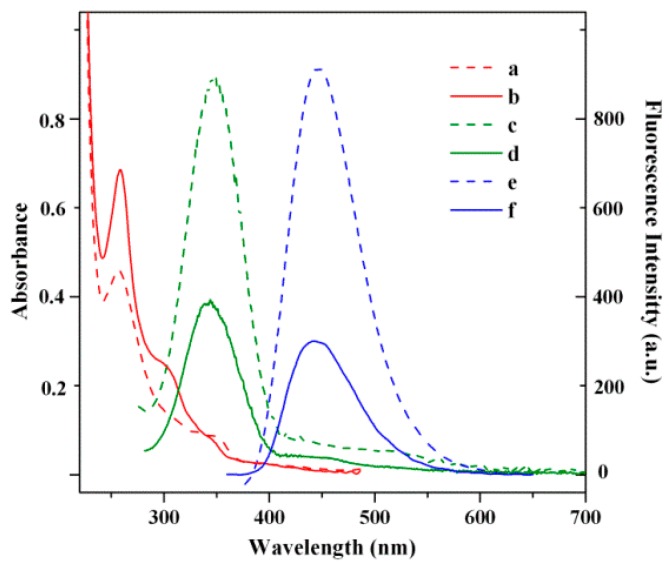
UV–vis (a, b), FL excitation (c, d) and FL emission spectra (e, f) of the FCD solution in the absence (a, c, e) and presence (b, d, f) of 50 μM Cu^2+^.

The quenching mechanism can be explained as follows. As mentioned above, the introduction of Cu^2+^ into the FCDs results in a new UV absorbance band centered at 306 nm. Notably, the solution of Cu^2+^ shows almost no absorption peak above 250 nm (Figure S4), while such a new absorption band can also appear when 140 μM Cu^2+^ was added into the PPDA solution. Considering that the synthesized FCDs are PPDA-capped, we speculate that the new absorbance peak can be attributed to the addition of Cu^2+^ with the PPDA on the surface of FCDs. Moreover, the absorbance band located at 306 nm (Figure 5 line b) partially overlapped with the excitation spectrum of the FCDs (Figure 5 line c). The overlapping between the absorption band of an absorber (in this case, the PPDA–Cu^2+^ complex) and the FL excitation (and/or emission) spectrum of the fluorophores (in this case, FCDs) can result in a decrease of the FL intensity of the fluorophore, which is the so-called inner filter effect (IFE) [24]. In short, the quenching of the FCDs’ FL by Cu^2+^ ions may be attributed to the formation of PPDA–Cu^2+^ complexes at the surface of FCDs that can absorb the excitation light of FCDs; in other words, the addition of Cu^2+^ decreases the concentration of the fluorescent FCDs.

### 3.4. Selective Detection of Cu^2+^ in Water

**Figure 6:**
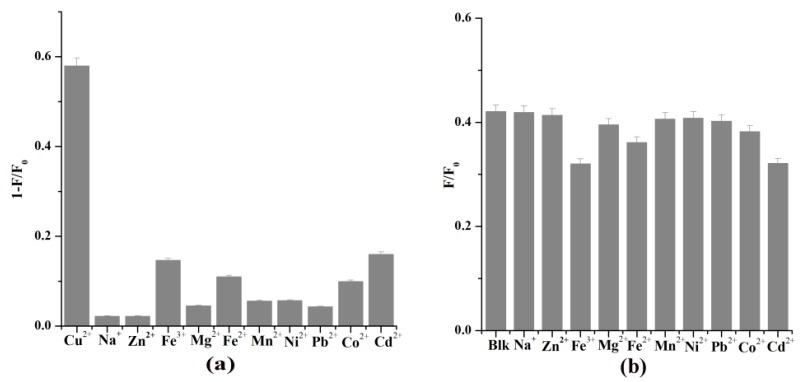
(**a**) FL intensity changes (*F*/*F*_0_) of FCDs upon addition of various metal ions (10 μM). (**b**) The effect of coexisting metal ions (10 μM) on selective sensing of Cu^2+^ by FCDs (Blk is only 10 µM Cu^2+^, λ_ex_ = 320 nm, λ_em_ = 452 nm).

To assess the selectivity of FCDs toward Cu^2+^, the quenching effect of other metal cations (Zn^2+^, Na^+^, Fe^3+^, Mg^2+^, Fe^2+^, Mn^2+^, Ni^2+^, Pb^2+^, Co^2+^, and Cd^2+^), as well as the mixture of Cu^2+^ and one of the above cations were investigated. The addition of Cu^2+^ resulted in apparent PL quenching, while other metal cations induced quite a few changes in the FL intensity of the FCDs (Figure 6a). Furthermore, the coexistence of other cations did not cause obvious interference for FCDs when sensing Cu^2+^ (Figure 6b) and allowed for the highly selective detection of Cu^2+^.

It should be noted here that 1,10-phenantroline is a commonly used reagent for the determination of Fe^3+^. However, the FL intensity of the FCDs was not significantly quenched with the addition of Fe^3+^. We then examined the effect of Fe^3+^ ions on the absorption spectra of the FCDs. As can be seen from Figure S5, under the addition of Fe^3+^, the absorption bands at 350 nm increased slightly, and no new peak appeared. This phenomenon may illustrate the weak interaction between Fe^3+^ and the FCDs, which may be responsible for the weak quenching effect of Fe^3+^ on the FL intensity of FCDs.

### 3.5. S^2−^ Detection by FCD–Cu^2+^

**Figure 7:**
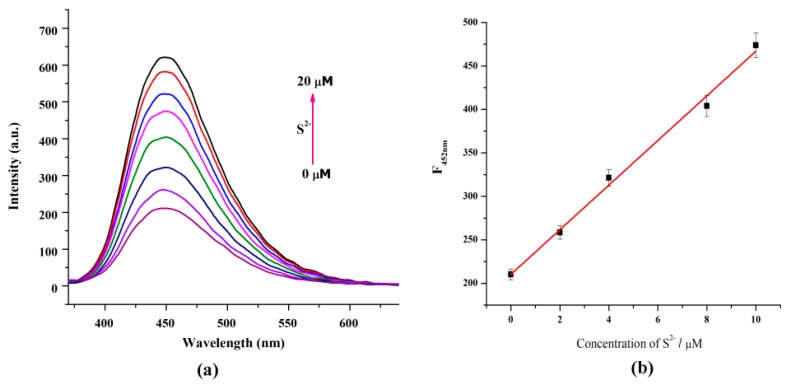
(**a**) FL response of the FCD–Cu^2+^ system (20 µM of Cu^2+^ in dilute FCD dispersion) upon the addition of S^2−^ (0–20 µM, λ_ex_ = 320 nm). (**b**) Plot of FL intensity of FCD–Cu^2+^ against S^2−^ concentration, λ_ex_ = 320 nm, λ_em_ = 452 nm.

Since S^2−^ can react with Cu^2+^ and generate a very stable CuS species [22], the addition of S^2−^ into the FCD–Cu^2+^ complex may result in the release of FCDs, which can make the FCD–Cu^2+^ possible to use as a turn-on FL sensor for S^2−^. Based on the above considerations, fluorescent titration was carried out to assess the FL response of the FCD–Cu^2+^ complex to S^2−^. As delineated in Figure 7a, the FL intensity of the FCD–Cu^2+^ complex was gradually enhanced after the addition of S^2−^.

The FL intensity increased nearly three-fold when the S^2−^ concentration was 20 µM. The FL intensity of FCD–Cu^2+^ and the S^2−^ concentrations show a linear relationship in the range of 0–10 µM (Y = 210.3426 + 25.6692X, *R*^2^ = 0.9971, Figure 7b). The detection limit of the FCD–Cu^2+^ for S^2−^ was determined to be 88.9 nM, which is clearly below the recommended maximum S^2−^ concentration in drinking water (about 15 µM) [31]. Therefore, the sensor can be used for S^2−^ detection in water quality monitoring.

**Figure 8:**
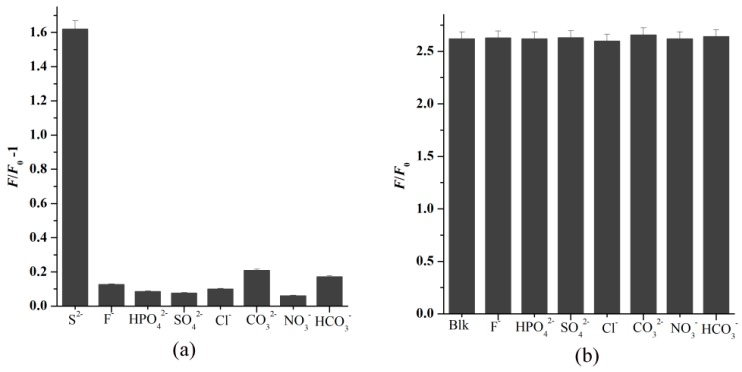
(**a**) FL response of FCD–Cu^2+^ upon addition of various anions (15 µM). (**b**) The effect of various coexisting anions (15 µM) on selective sensing of S^2−^ by FCD–Cu^2+^ system (Blk is 20 µM Cu^2+^ and 15 µM S^2−^, λ_ex_ = 320 nm, λ_em_ = 452 nm).

To study the selectivity of FCD–Cu^2+^ toward S^2−^, the quenching effect of other anions (F^−^, Cl^−^, HCO_3_^−^, SO_4_^2−^, NO_3_^−^, CO_3_^2−^, and HPO_4_^2−^), the mixture of S^2−^, and one of the above anions were investigated. Only the introduction of S^2−^ can result in the apparent PL enhancement of FCD–Cu^2+^ (Figure 8a), exhibiting outstanding selectivity for S^2−^ detection. Moreover, the coexistence of other anions shows a negligible effect on the FL enhancement of the FCD–Cu^2+^ complex (Figure 8b), which indicates that FCD–Cu^2+^ can be used as an effective sensor for S^2−^ with high selectivity.

### 3.6. Intracellular FL imaging of Cu^2+^ and S^2−^

**Figure 9:**
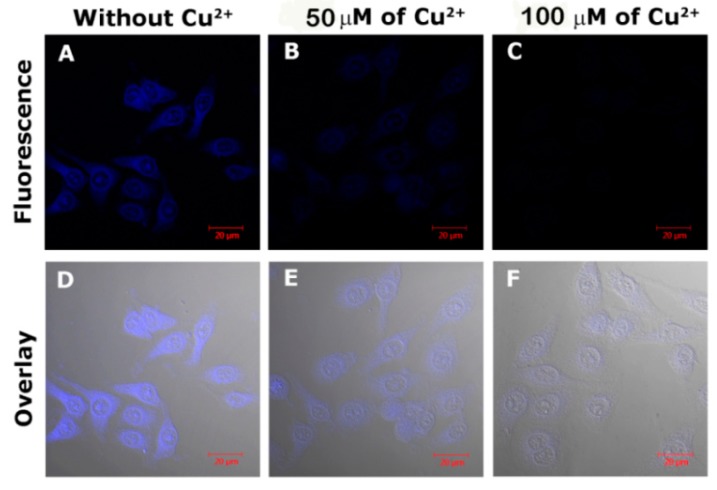
Confocal images of HeLa cells incubated with FCDs sensor (FCD, 50 µg mL^−1^, **A**,**D**) and treated with 50 µM (**B**,**E**) and 100 µM (**C**,**F**) of Cu^2+^. The overlay images confirm the signals are from in-cell FCDs, not interference.

**Figure 10:**
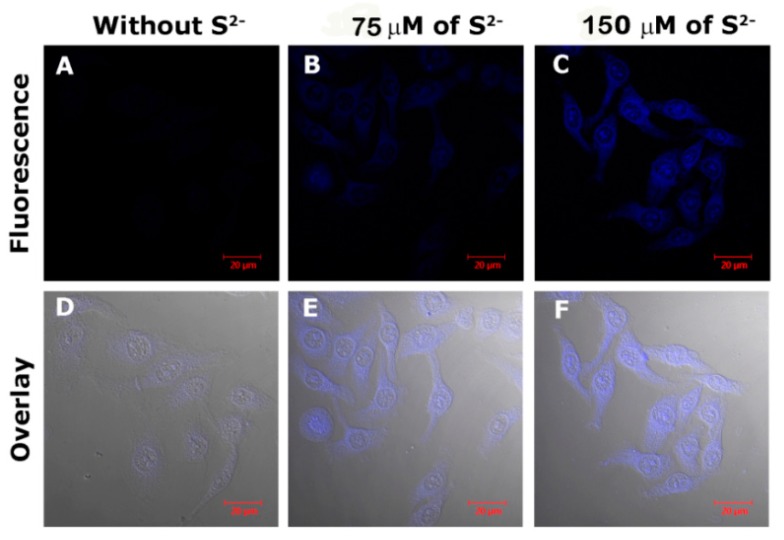
Confocal images of HeLa cells incubated with the FCD sensor (FCD, 50 µg mL^−1^) and 100 µM of Cu^2+^ (**A**,**D**), and then treated with 75 µM (**B**,**E**) and 150 µM of S^2−^ (**C**,**F**). The overlay images confirm the signals are from in-cell FCDs, not interference.

To evaluate the feasibility of FCDs for Cu^2+^ imaging in live cells, HeLa cells were chosen to be incubated with the FCDs. As depicted in Figure 9, after the introduction of Cu^2+^ to the HeLa cells, the FL intensity of the FCDs decreased significantly and almost disappeared with 100 µM of Cu^2+^. Furthermore, the ability of the FCD–Cu^2+^ complex to monitor intracellular S^2−^ was also tested. HeLa cells were firstly incubated with the FCD–Cu^2+^ and subsequently treated with S^2−^. As shown in Figure 10, with the increasing S^2−^, the FL intensity of the cell also clearly increased (recovered). These results indicate the great potential of FCDs and FCD–Cu^2+^ for monitoring Cu^2+^ and S^2−^ in live cells.

### 3.7. Photostability of FCDs

The as-synthesized FCDs exhibited prominent photostability under different conditions. The FL intensity of the FCDs solution remained almost unchanged under continuous excitation (80 min) using 365 nm UV light (Figure S6). Moreover, when the FCDs were stored for 40 days, negligible changes (<3%) in the *F*/*F*_0_ intensity of the FCDs in the presence of Cu^2+^ (15 µM) were observed (Figure S7). No obvious F/F_0_ changes of the FCDs were noticed in the biologically-related pH range 5.0–9.0 after the addition of small concentrations of Cu^2+^ (Figure S8). These results indicate that the FCDs possess favorable FL stability under light excitation, air conditions, and pH variation, and can be used for the detecting and imaging of Cu^2+^ in complex environments.

### 3.8. Analysis in a Real Sample

To further test the applicability of this sensing system for detecting Cu^2+^ in a real sample, we used a standard addition method to detect the concentration of Cu^2+^ ions in several tap water samples. As displayed in Table S2, the relative standard deviation (RSD) for all tests are less than 0.38% while recoveries were in the range from 85% to 99.5%. These results indicated the high precision and accuracy of this CD-based FL sensing method for the detection of Cu^2+^ in real samples.

## 4. Conclusion

In summary, a novel fluorescent nanoprobe was elaborately fabricated by grafting a phenanthroline derivative onto the surface of CDs. This nanoprobe exhibits good selectivity and sensitivity (detection limit: 40.1 nM) for Cu^2+^ detection due to a specific inner filter effect. The generated FCD–Cu^2+^ complex can be further used as an effective off–on-type sensor for the detection of S^2−^ with a detection limit of 88.9 nM. Furthermore, the FCD sensor with excellent photostability can also be applied for the monitoring intracellular Cu^2+^ and S^2−^. These CDs-based nanoprobe may have great prospects in the biological field in the future. This work may provide some meaningful insights for constructing CD-based multifunctional FL sensors to detect analytes in the biological and environmental fields.

## Supplementary Materials

The following are available online at http://www.mdpi.com/2079-4991/8/12/1071/s1, Figure S1: The UV–vis and fluorescence emission spectra of the pristine CDs (λ_ex_ = 360 nm); Figure S2: The fluorescence emission spectra of CDs under different excitation wavelength (λ_ex_ = 280~460 nm); Figure S3: Fluorescence titration of the un-functionalized CDs sample with different Cu^2+^ concentrations (λ_ex_ = 320 nm); Figure S4: UV spectra of 140 μM Cu^2+^ ions, 100 μM PPDA, and 140 μM Cu^2+^ ions + 100 μM PPDA; Figure S5: UV spectra of FCDs upon addition of Fe^3+^; Figure S6: Fluorescence intensity changes of the FCDs under a continuous 365 nm UV lamp irradiation (λ_ex_ = 365 nm, λ_em_ = 452 nm); Figure S7: Longterm photostability of FCDs dispersion for Cu^2+^ recognition (Cu^2+^ concentration: 15 μM, λ_ex_ = 320 nm, λ_em_ = 452 nm); Figure S8: Fluorescence intensity changes of the FCDs under pH range 5.0–9.0 upon addition of Cu^2+^ (Cu^2+^ concentration: 10 μM, λ_ex_ = 320 nm, λ_em_ = 452 nm); Table S1: Comparison of representative CDs-based sensors for Cu^2+^ or/and S^2−^ detection; Table S2: Determination of Cu^2+^ in tap water.
