# Supplementary material for: Phenanthroline-Derivative Functionalized Carbon Dots for Highly Selective and Sensitive Detection of Cu2+ and S2− and Imaging inside Live Cells
Source: Nanomaterials (Basel). 2018 Dec 19;8(12):1071. doi: 10.3390/nano8121071 (PMC6315650; doi:10.3390/nano8121071)
Supplement: Supplementary file 1 [file nanomaterials-08-01071-s001.pdf]

Supplementary information

Phenanthroline-Derivative Functionalized Carbon Dots for Highly Selective and Sensitive Detection of Cu^{2+} and S^{2-} and Imaging inside Live Cells

Lina Zhang [1,2,*], Zhanwei Wang [1], Jingbo Zhang [1], Jianbo Jia [1], Dan Zhao [1] and Yunchang Fan [1,*]

[1] College of Chemistry and Chemical Engineering, Henan Polytechnic University, Jiaozuo 454003, China; 13290700557@163.com (Z.W.); zhangjb5464@163.com (J.Z.); jiajianbo@hpu.edu.cn (J.J.); iamzd@hpu.edu.cn (D.Z.)

[2] Henan Key Laboratory of Coal Green Conversion, Jiaozuo 454003, China

* Correspondence: zhln@hpu.edu.cn (L.Z.); fanyunchang@hpu.edu.cn (Y.F.); Tel.: +86-10-0391-3986813 (Y.F.)

Table S1. Comparison of representative CDs-based sensors for Cu^{2+} or/and S^{2-} detection.

**Sensors**	**Detected ion**	**Detection limit (nM)**	**Comments**	**Reference.**
CdSe@CDs-TPEA	Cu^{2+}	1000	High selectivity; Imaging in living cell; Complexity in synthesis.	Zhu et al. Angew. Chem.-Int. Ed., 51 (2012) 7185-7189.
Europium complex functionalized CDs	Cu^{2+}	4	High selectivity; High cost.	Ye et al. New J. Chem., 38 (2014) 5721-5726.
CDs-TPEA	Cu^{2+} and S^{2-}	700 for S^{2-}	Turn-on for S^{2-} High selectivity; Imaging in living cell.	Zhu et al. Analyst, 139 (2014) 1945-1952.
naphthalimide-functionalized CDs	S^{2-}	10	Good selectivity; Detection in water–ethanol solution; Imaging in living cell.	Yu et al. Chem. Commun., 49 (2013) 403-405.
Ligand existing CDs	Cu^{2+} and S^{2-}	Cu^{2+}: 1720, S^{2-}: 780	Good selectivity; Imaging in living cell.	Hou et al. Nanotechnology, 24 (2013) 335502.
Cyclam-capped CDs	Cu^{2+} and S^{2-}	Cu^{2+}: 100, S^{2-}: 130	Good selectivity; Renewable sensing; fluorescence stability; Imaging in living cell.	Chen et al. Sens. Actuators B. **2016**, 224, 298–306
PPDA-functionalized CDs	Cu^{2+} and S^{2-}	Cu^{2+}: 40.1, S^{2-}: 88.9	Good selectivity; Renewable sensing; fluorescence stability; Imaging in living cell.	This work.

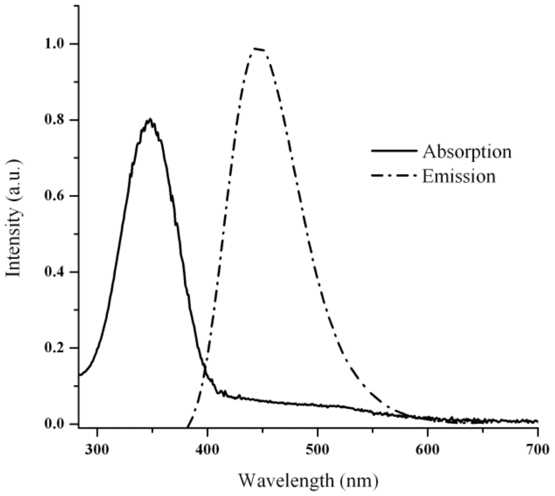

Figure S1. The UV–vis and fluorescence emission spectra of the pristine CDs (λ_{ex} = 360 nm).

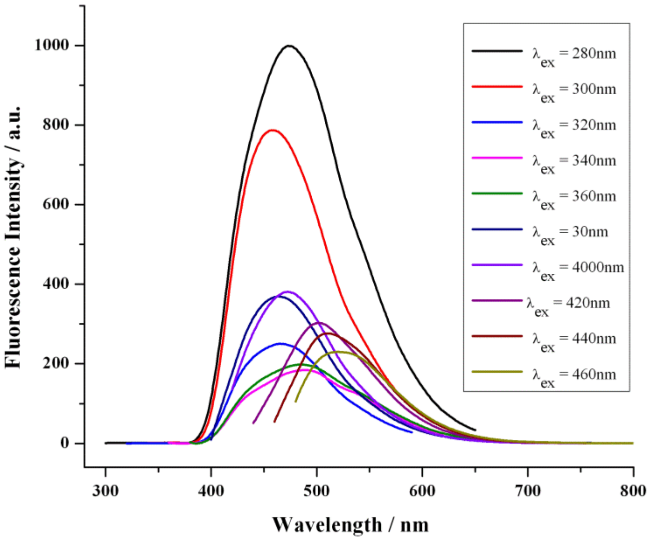

Figure S2. The fluorescence emission spectra of CDs under different excitation wavelength (λ_{ex} = 280~460 nm).

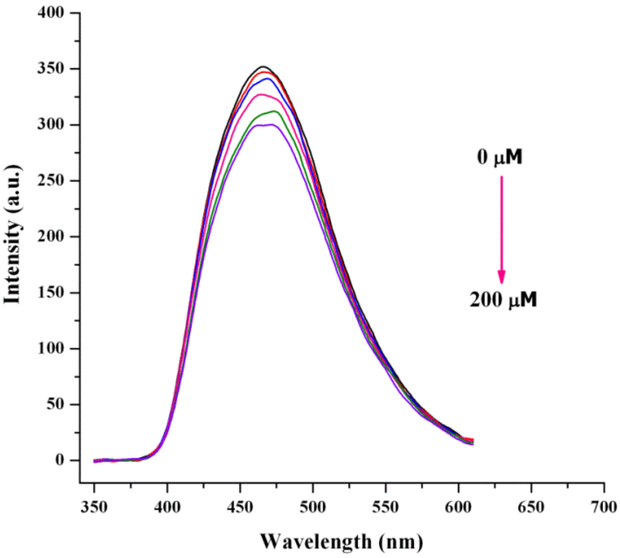

Figure S3. Fluorescence titration of the un-functionalized CDs sample with different Cu^{2+} concentrations (λ_{ex} = 320 nm).

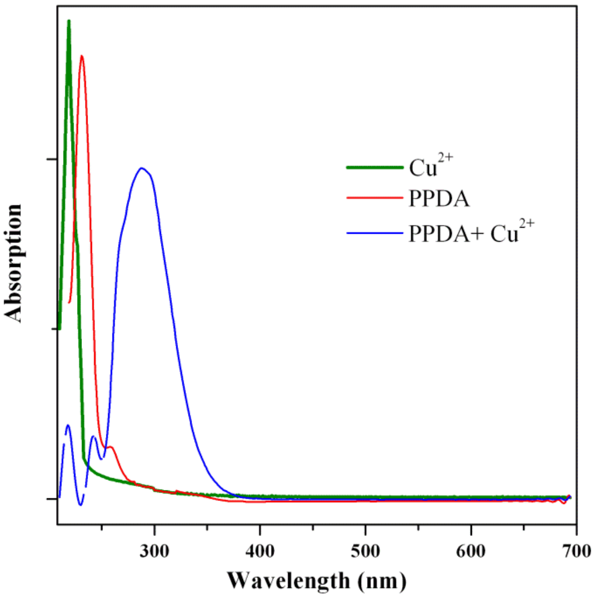

Figure S4. UV spectra of 140 μM Cu^{2+} ions, 100 μM PPDA, and 140 μM Cu^{2+} ions + 100 μM PPDA.

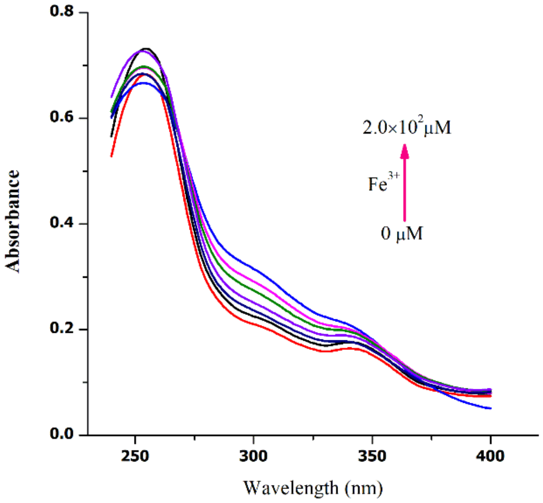

Figure S5. UV spectra of FCDs upon addition of Fe^{3+}.

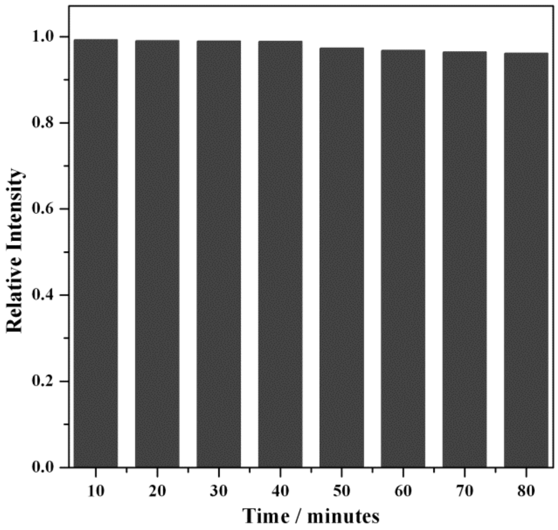

Figure S6. Fluorescence intensity changes of the FCDs under a continuous 365 nm UV lamp irradiation (λ_{ex} = 365 nm, λ_{em} = 452 nm).

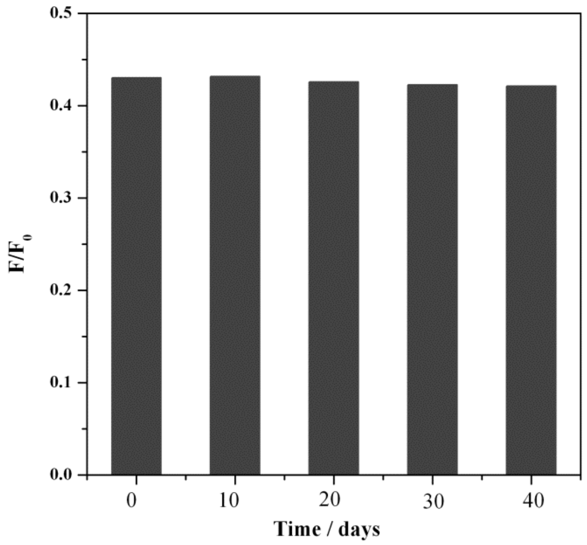

Figure S7. Longterm photostability of FCDs dispersion for Cu^{2+} recognition (Cu^{2+} concentration: 15 μM, λ_{ex} = 320 nm, λ_{em} = 452 nm).

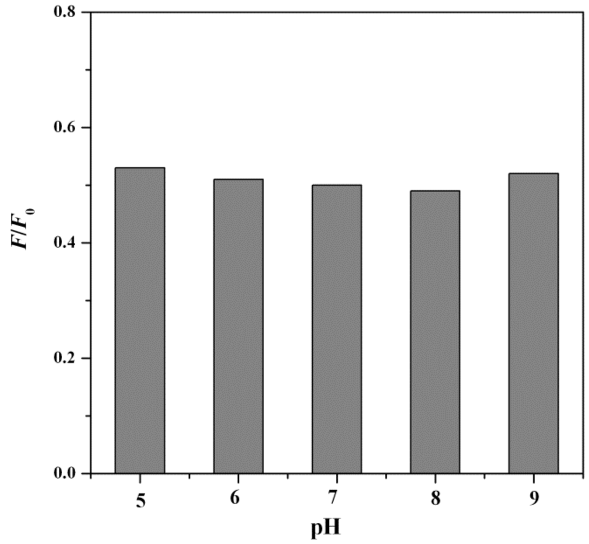

Figure S8. Fluorescence intensity changes of the FCDs under pH range 5.0–9.0 upon addition of Cu^{2+} (Cu^{2+} concentration: 10 μM, λ_{ex} = 320 nm, λ_{em} = 452 nm).

Table S2. Determination of Cu^{2+} in tap water.

samples	Added amount of Cu^{2+}	Found amount of Cu^{2+}	RSD (n = 3, %)	Recovery (%)
Tap water	3.00	2.97	0.34	99.0
	4.00	4.05	0.25	101.2
	5.00	4.96	0.20	99.2
